# Functional Diversity of Wintering Waterbird Enhanced by Restored Wetland in the Lakeshore of Chaohu Lake

**DOI:** 10.1002/ece3.71751

**Published:** 2025-07-07

**Authors:** Shanshan Xia, Xianglin Ji, Lei Meng, Lizhi Zhou

**Affiliations:** ^1^ School of Resources and Environmental Engineering Anhui University Hefei China; ^2^ Anhui Province Key Laboratory of Wetland Ecosystem Protection and Restoration, Anhui University Hefei China

**Keywords:** environmental filtering, functional diversity, gate‐controlled lake, lakeshore wetland, restored wetland, waterbird diversity

## Abstract

The primary goal of wetland restoration is to understand the species assembly process in order to strategically guide community development. Providing suitable habitats for waterbirds is one of the key objectives. In recent years, Chaohu Lake—a typical gate‐controlled reservoir lake in the middle and lower Yangtze River floodplain—has faced wetland habitat homogenization due to sustained high‐ water‐ levels. This has made the maintenance of regional waterbird metacommunity diversity a critical concern. This study employed a trait‐based approach, using Linear Mixed Models (LMMs) and Generalized Dissimilarity Modeling (GDM), to quantify the impacts of lakeshore wetland restoration (2020–2023) on wintering waterbird functional diversity. Results showed significant enhancements in functional α‐diversity within restored wetlands (FRic, FDis, and FDiv, *p* < 0.01), highlighting the importance of habitat heterogeneity. Notably, restored wetlands exhibited coupled patterns of higher functional β‐diversity turnover rates and lower functional nestedness over time, forming distinctive species assemblages. Environmental filtering was primarily driven by trait loss mechanisms, with water depth and aquatic vegetation coverage identified as key drivers. In conclusion, wetland restoration significantly enhanced the functional diversity of wintering waterbirds in Chaohu Lake, emphasizing the critical role of functional diversity in evaluating restoration effectiveness for high‐ water‐ level gate‐controlled floodplain lakes.

## Graphical abstract

A trait‐based approach was used to quantify wintering waterbirds in a gate‐controlled floodplain lake. Local community composition of a unrestored wetland is a characteristic subset of the restored wetlands. Environmental filtering leads to functional differences in the gate‐controlled floodplain lake. Functional diversity is critical to assess the effectiveness of wetland restoration.

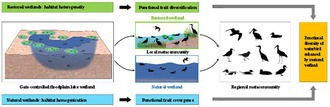

## 1 Introduction

Understanding community assembly mechanisms remains a central challenge in community ecology (Overcast et al. 2021). Metacommunity theory provides an integrative framework that reconciles local environmental filtering with regional dispersal processes across spatial hierarchies (Thompson et al. 2020). This paradigm posits that species sorting and dispersal limitation jointly determine community composition, with quantifying their relative contributions being a key objective in metacommunity ecology (Wu et al. 2018). Floodplain wetlands epitomize dynamic mosaics of habitat patches (Heng et al. 2018; Park and Latrubesse 2017), where nested waterbody patchs exhibit island‐like ecological patterns (Iskin and Wohl 2023). Within these ecosystems, waterbirds actively respond to patch heterogeneity through habitat selection, forming spatially structured metacommunities that mirror hydrological connectivity gradients.

Reasonable hydrological regulation is critical for maintaining wintering waterbird diversity in the middle and lower Yangtze River floodplains (Wei and Zhou 2023; Xia et al. 2017; Zhang et al. 2021). Regulating lake water levels by controlling gates and dams is the most typical hydrological disturbance (Qiu et al. 2021; Wang et al. 2020). These lakes exhibit three hydrological regimes: natural, intermittent, and reservoir‐regulated fluctuations (Xia et al. 2016). Reservoir‐type lakes maintain artificially elevated dry‐season water levels, resulting in an environmental filtering effect that homogenizes the foraging habitat heterogeneity of waterbirds to a certain extent. This directly affects the abundance and availability of their food resources—particularly suppressing small wading species with short tarsus lengths—and poses key challenges to metacommunity diversity conservation in gate‐controlled wetlands (Quiroga et al. 2021). Restoring heterogeneous habitats through targeted eco‐engineering is therefore imperative for enhancing regional biodiversity.

Wetland restoration is an effective approach to biodiversity conservation (O'Brien et al. 2022), aiming to guide ecosystem development through an understanding of community assembly processes to achieve and sustain high biodiversity (Hulvey et al. 2014; Wainwright et al. 2018). As bioindicators of wetland integrity, waterbird communities provide critical metrics for evaluating restoration outcomes (Casazza et al. 2021; Fan et al. 2021; He et al. 2023). Current restoration efforts in gate‐controlled reservoir floodplain wetlands focus on constructing ecological barriers by creating patchy wetland mosaics to mitigate environmental filtering effects caused by sustained high water levels. However, the ecological benefits of such restoration remain uncertain. While traditional assessments prioritized taxonomic diversity recovery (e.g., species‐specific population restoration or pre−/postrestoration species richness comparisons) (Kačergytė et al. 2022; Zhou et al. 2020), modern restoration paradigms emphasize ecological function enhancement (Htay et al. 2023), necessitating evidence‐based approaches grounded in functional diversity analyses integrated with freshwater ecology and conservation biology principles (O'Brien et al. 2022).

In recent years, functional diversity, a key focus in community ecology (Gauzere et al. 2022; Mammola et al. 2021), has quantified species trait‐environment adaptations to elucidate community assembly mechanisms (Cadotte et al. 2013; Munoz et al. 2023; Vernham et al. 2023). Its alpha (average species richness per site) and beta (functional turnover/nestedness between sites) components disentangle ecological processes—environmental filtering, dispersal limitation, and competition—during restoration (Chao et al. 2019; Coccia et al. 2021). High functional alpha diversity supports broader ecological functions (Cadotte et al. 2011), while beta components guide spatially tailored conservation strategies (Ni et al. 2020). By linking diversity to habitat functionality (Derhé et al. 2018), functional diversity is ideal for evaluating restoration success, yet remains underutilized. Waterbirds' trait‐based habitat preferences reflect wetland restoration quality, necessitating a functional trait framework to compare community differences across wetland types.

Chaohu Lake, a typical gate‐controlled reservoir lake in the middle and lower Yangtze River floodplain, has experienced intensive hydrological regulation, leading to habitat homogenization in its lakeshore wetlands due to sustained high water levels. This homogenization has significantly degraded the natural mudflat‐meadow gradient heterogeneity characteristic of floodplain wetland systems. To restore and maintain biodiversity, a wetland restoration project (including ten wetland parks, completed in 2023) was implemented around Chaohu Lake in 2020. This project employed hydrological connectivity restoration and heterogeneous vegetation configuration to mitigate habitat filtering effects, establishing a paradigm for restoration studies in gate‐controlled lake wetlands. Guided by metacommunity theory, this study integrates functional trait‐based approaches and β‐diversity decomposing to quantify structural differences in overwintering waterbird communities between restored wetlands and unrestored high‐water‐level wetlands (anthropogenically homogenized habitats) across the lakeshore. We further elucidate mechanisms underlying functional diversity responses to wetland restoration under dry‐season high‐water‐level regulation. The following hypotheses were tested: (a) Wetland type (restored vs. unrestored) dominates functional α diversity, with restored wetlands exhibiting significantly higher diversity due to enhanced habitat heterogeneity. (b) Restored wetlands disproportionately contribute to regional functional β diversity through turnover components driven by strengthened niche complementarity, whereas unrestored wetlands primarily influence nestedness components via habitat filtering. (c) Environmental differences between restored and unrestored wetlands drive the functional diversity and its components.

## 2 Materials and Methods

### 2.1 Study Area

The Chaohu Lake basin, encompassing an area of ~13,500 km^2^, constitutes an important part of the water network ecosystem in the middle and lower Yangtze River floodplain. Characterized by a radial pattern of inflowing rivers centered around Chaohu Lake, the basin's hydrological system ultimately connects to the Yangtze River through the Yuxi River. The construction of three major sluices (Chaohu, Zhaohe, and Yuxi) has transformed the lake into an artificially regulated reservoir (Ren et al. 2021). Consequently, persistent high water levels maintained throughout the year, primarily due to shipping activities and other anthropogenic factors, have significantly impaired the lake's ecological functions. These hydrological alterations have led to a substantial reduction in habitat heterogeneity and diminished food resource availability for waterbirds, thereby threatening the basin's biodiversity and ecosystem stability.

**FIGURE 1:**
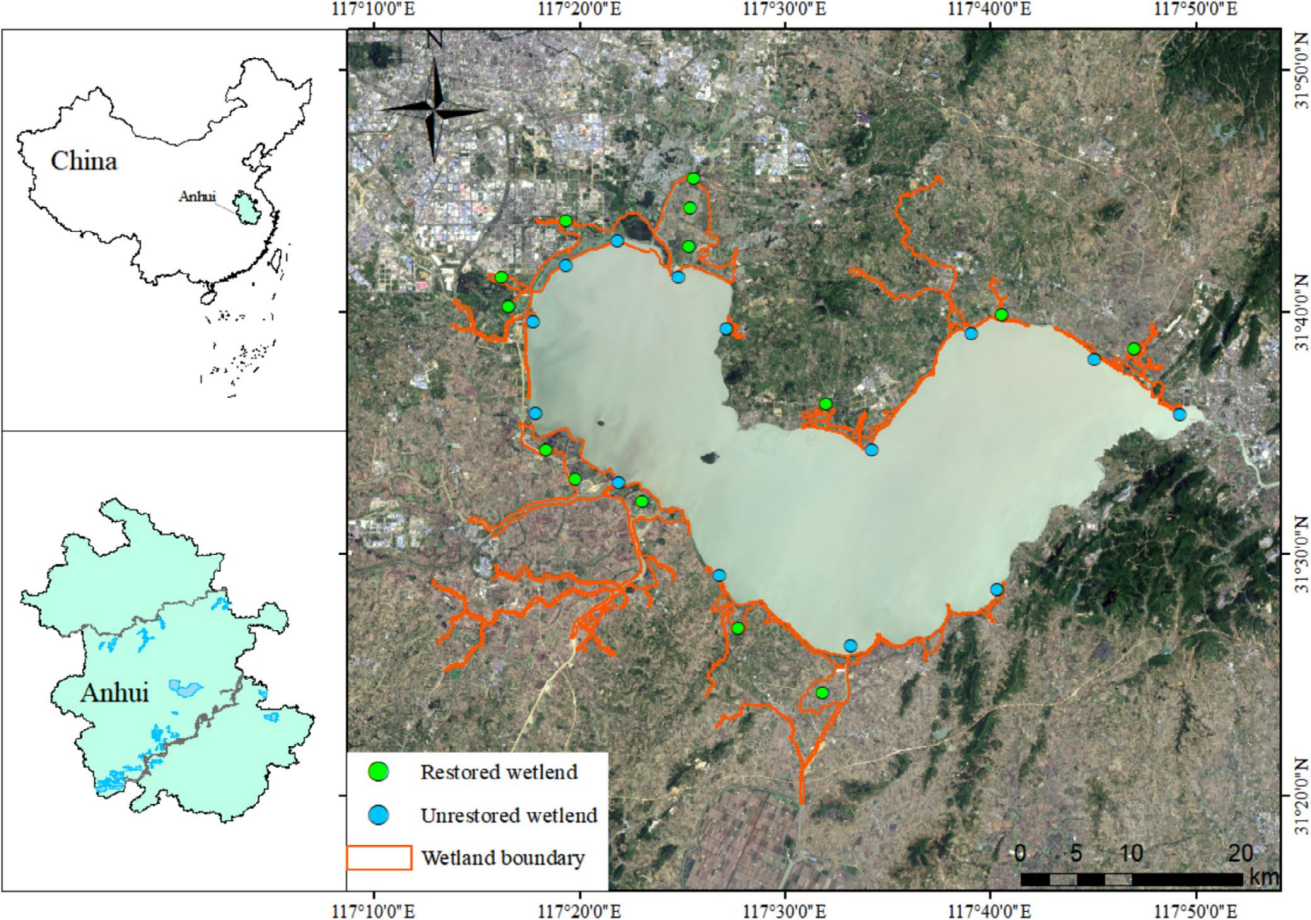
Sampling points and areas in the local community of Chaohu Lake, Anhui Province, China.

The wetland restoration initiative surrounding Chaohu Lake has been systematically implemented through the establishment of 10 major wetland parks along the lakeshore zones. These parks incorporate dual functionalities of floodwater retention and vegetation configurations specifically designed to adapt to seasonal water‐level fluctuations. This integrated approach aims to create ecological buffer zones dominated by wetland ecosystems to serve as a strategic measure to mitigate lakeshore non‐point source pollution. Within the meta‐ecosystem framework, restored wetlands are strategically embedded as patches within a meta‐ecosystem framework, while unrestored wetlands represent lakeshore areas in the gate‐controlled floodplain that have maintained their original state despite long‐term anthropogenic impacts, exhibiting managed homogenized habitats. To evaluate the ecological outcomes of this restoration initiative, we conducted a comparative study of waterbird diversity between restored and unrestored wetlands within the gate‐controlled floodplain lake system, thereby establishing paired comparisons of managed and reference wetland ecosystems (Figure 1).

### 2.2 Taxonomic and Environmental Data

A comprehensive sampling strategy was implemented, with 28 sample points strategically distributed across two distinct wetland types (14 restored and 14 unrestored points). These points represent local metacommunities that collectively form a regional metacommunity within the gate‐controlled floodplain lake wetland complex. Sampling was conducted at 28 permanent observation points during the wintering seasons (December to February) over three consecutive years (2020–2023).

Waterbird counts were obtained using standardized observation techniques with high‐quality optical equipment, including binoculars (Swarovski, 8.5 × 42, Austria) and monoculars (Swarovski, ATM 20–60 × 85). A fixed‐point counting method was employed, with each sampling point covering a semi‐circular area of approximately 39 ha (radius = 500 m). To ensure spatial independence and comprehensive coverage, sampling points were georeferenced using latitude and longitude grids, maintaining a minimum distance of 1 km between adjacent points (Aguilar et al. 2021). Observations were conducted during 15–20‐min periods, during which all birds within the designated area (excluding overflying individuals) were identified and counted (Woldemariam et al. 2018). Field surveys were restricted to daylight hours under optimal weather conditions (Table S1) with precise GPS coordinates recorded at each site using a GPS (eTrex30; Garmin, China) handheld device (Figure 1).

To assess the drivers of functional diversity in waterbirds, we analyzed two sets of explanatory factors (Table S2): Environmental Variables: (a) Habitat Attributes: Aquatic vegetation cover (ACV), water depth (WD); (b) Water Quality Parameters: pH, dissolved oxygen (DO), water transparency (TD), water temperature (WT), electrical conductivity (EC); (c) Anthropogenic Disturbances: Distance to roads (RD), distance to villages (VD), human disturbance intensity (HI). These variables were selected based on their well‐documented influence on species diversity (Matuoka et al. 2020; Schaffer‐Smith et al. 2018; Woldemariam et al. 2018). Spatial Variables: Geographic distances between sample sites were calculated using latitude and longitude coordinates to model spatial processes related to bird dispersal dynamics.

This methodological framework enabled a comprehensive analysis of both environmental filtering and spatial processes in shaping waterbird communities within the study area.

### 2.3 Functional Traits and Functional Diversity

#### 2.3.1 Functional Trait Selection and Characterization

We utilized the Global Bird Trait Dataset (Tobias et al. 2022) to extract 21 functional traits across three categories: morphological, ecological, and geographical characteristics (Table S3). The selected traits included:

Morphological Traits (11) include Beak morphology (4 measurements), Wing characteristics (3 measurements), Tarsal length, Tail length, Wing index, and Body mass.

Ecological Traits (5) include Trophic characteristics: Trophic level (herbivorous, carnivorous, omnivorous) and Trophic niche (aquatic predator, aquatic herbivore); Habitat preferences: Habitat type (lakes, reed thickets) and Habitat characteristics (dense, semi‐open, open), and Migration patterns.

Geographical Traits (5) include Species range (km^2^), Centroid longitude, Latitudinal distribution (maximum, minimum, centroid).

These traits collectively provide comprehensive information on species' ecological niches and phenotypic characteristics, particularly those related to feeding ecology, dispersal capacity, and movement patterns.

#### 2.3.2 Functional α‐Diversity Quantification

We assessed functional α‐diversity using presence–absence data across multiple wintering periods through four complementary indices (Almeida et al. 2019): Functional Richness (FRic) quantifies the volume of functional space occupied by species traits, reflecting resource‐use potential; Functional Evenness (FEve) measures the uniformity of trait distribution within functional space; Functional Divergence (FDiv) evaluates the deviation of trait distribution from the functional space centroid; Functional Dispersion (FDis) represents the average functional dissimilarity among species.

The analysis involved: computation of Gower distances to generate functional trait distance matrices; identification of optimal functional space dimensions through Principal Coordinates Analysis (PCoA) and quality assessment (Figure S1); retention of the first four PCoA axes (explaining 89% variance) and exclusion of samples with < 4 species and transient species (single‐recorded individuals).

Community Weighted Mean (CWM) values (Gaüzère et al. 2019) were calculated as abundance‐weighted trait means to compare trait differences between wetland types, reflecting environmental filtering effects. Key traits driving functional space differentiation were identified through correlation analysis between CWM and PCoA axes (excluding traits with *r* < 0.6). All analyses were implemented using the “mFD” R package (Magneville et al. 2021), which provides standardized workflows for functional diversity analysis.

#### 2.3.3 Functional β‐Diversity Assessment

We quantified functional β‐diversity and its components (turnover and nestedness) across local and regional wetland communities using: Total Functional β‐Diversity: Measured using the Sørensen index to represent overall functional dissimilarity; Component Decomposing: Decomposition of β‐diversity into turnover and nestedness components (Baselga and Orme 2012). Analytical procedures were conducted using: ’beta.pair’ function from the “betapart” package; betadisper function from the “vegan” package and R version 4.1.3 environment.

### 2.4 Data Analyses

To evaluate the impacts of wetland types on functional α diversity, we employed a comprehensive analytical approach. For each functional diversity index—including FRic (Functional Richness), FEve (Functional Evenness), FDiv (Functional Divergence), FDis (Functional Dispersion), and CWMs (Community Weighted Means) of highly correlated functional characteristics derived from PCoA axes—we constructed separate Linear Mixed Models (LMMs). This modeling framework was particularly suitable as it accommodates random effects, effectively addressing potential non‐independence among samples (Iyit and Genc 2011).

In our model specification, wetland type was implemented as a fixed effect to examine its direct influence on functional diversity metrics. To account for temporal pseudoreplication across different sampling periods, we incorporated wintering year as a random intercept term. Model selection and evaluation were performed through rigorous comparison between the full model and its corresponding null model (intercept‐only version) using the Akaike Information Criterion (AIC), a robust method for model comparison.

The explanatory power of each model was quantitatively assessed using the ‘r.squared GLMM’ function from the “MuMIn” package, which provides two key metrics: (a) marginal *R* ^2^ (*R* ^2^_m_), representing the variance explained solely by fixed effects, and (b) conditional *R* ^2^ (*R* ^2^_c_), indicating the combined explanatory power of both fixed and random effects. To determine the statistical significance of fixed effects, we conducted Likelihood Ratio Tests (LRTs) following the methodology established by Nakagawa and Schielzeth (2013), ensuring robust hypothesis testing within our mixed‐effects modeling framework. This comprehensive analytical approach allowed us to rigorously evaluate the effects of wetland types on various dimensions of functional diversity while appropriately accounting for temporal dependencies in our dataset.

To address the observed non‐normality and heteroscedasticity in our dataset, we implemented a stratified analytical approach. Specifically, we conducted separate analyses for each wintering year and utilized the non‐parametric Mann–Whitney *U* test to compare β‐diversity and its constituent components of wintering waterbird communities between restored and unrestored wetlands.

To elucidate the environmental drivers underlying functional β‐diversity patterns in restored wetlands, we employed the Generalized Dissimilarity Model (GDM) framework. The model incorporated both geographic distance matrices (derived from site coordinates) and Z‐score standardized environmental variables to predict β‐diversity configurations (Mokany et al. 2022). Our analysis focused on data from the 2022–2023 wintering season, following complete wetland restoration. The response variable was constructed using the phase dissimilarity matrix generated by the β.pair function.

The GDM implementation included several key methodological considerations: (a) three default I‐spline functions were specified to control the flexibility of nonlinear fitting; (b) model variance was quantified through the explanatory bias calculated by gdm.varImp; and (c) prediction accuracy was assessed using a dual‐metric approach combining *R* ^2^ (goodness‐of‐fit) and RMSE (error magnitude). Additionally, we employed Mantel tests to examine spatial autocorrelation characteristics between geographic distance and functional dissimilarity, with nonlinear response patterns visualized through I‐spline curves (Ferrier et al. 2007). All analyses were conducted in R (v 4.1.3).

## 3 Results

### 3.1 Functional Alpha Diversity Differences

**FIGURE 2:**
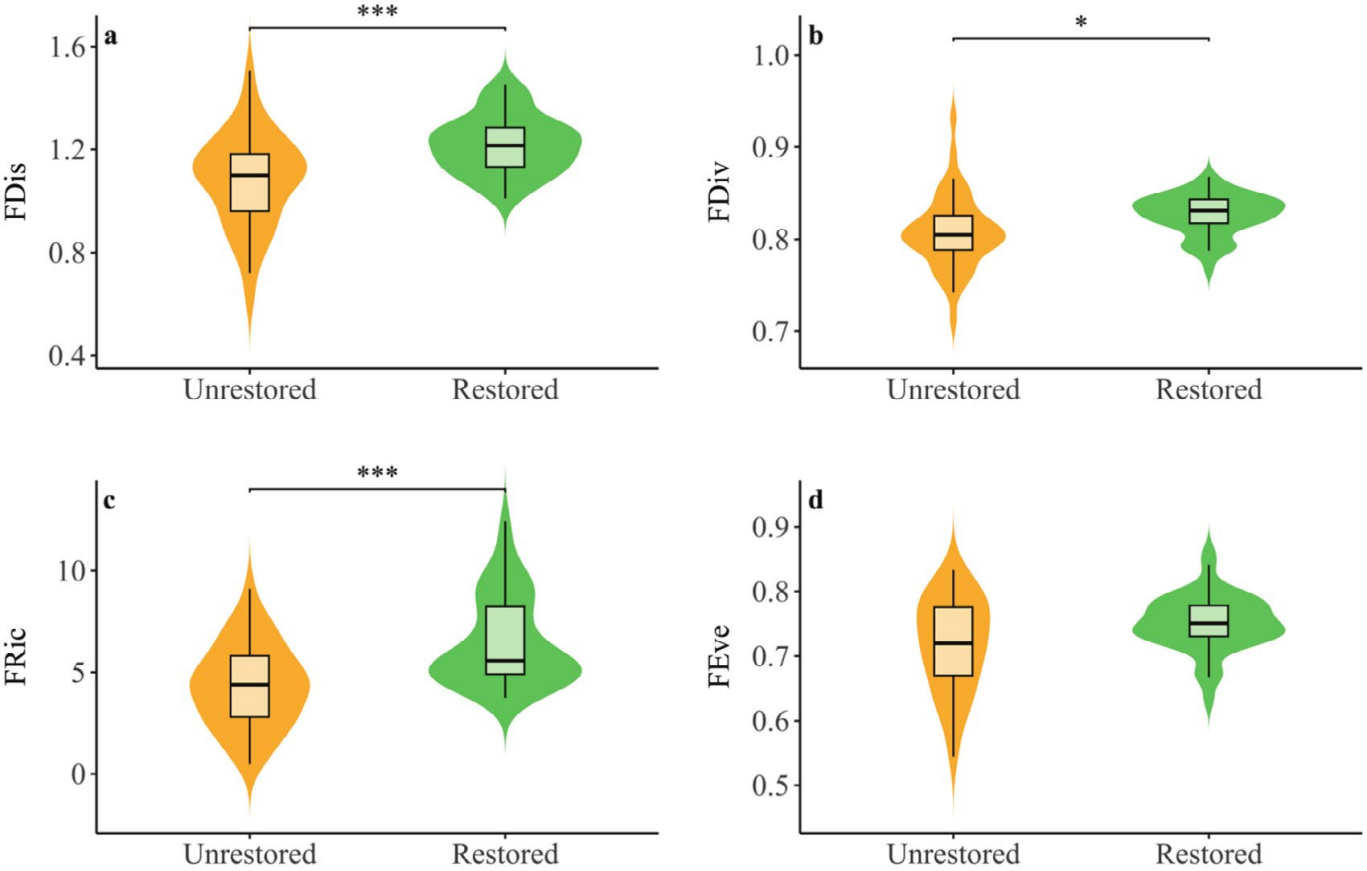
Plot showing the kernel probability density of the functional diversity index (mean ± SE) for α for different wetland types in the lakeshore wetlands in Chaohu Lake. Statistically significant values are represented by an asterisk sign: ***p < 0.001, *p < 0.05.

LMM showed that wetland restoration significantly enhanced the functional alpha diversity of wintering waterbirds in the lakeside of Chaohu Lake. Specifically, restored wetlands exhibited significantly higher FDis (ΔAIC = 13.8, *χ* ^2^ = 17.5, *p* < 0.001), FDiv (ΔAIC = 15.7, *χ* ^2^ = 5.6, *p* < 0.05), and FRic (ΔAIC = 18.7, *χ* ^2^ = 22.3, *p* < 0.001) compared to unrestored wetlands (Figure 2 and Table S4). Analyses of model explanatory power indicated that time‐series variation in wintering year had a small effect on FDis (*R* ^2^_c_ = 0.176, *R* ^2^_m_ = 0.173), FDiv (*R* ^2^_c_ = 0.196, *R* ^2^_m_ = 0.184), and FRic (*R* ^2^_c_ = 0.232, *R* ^2^_m_ = 0.219), and that habitat type in the fixed effects was a main drivers.

**FIGURE 3:**
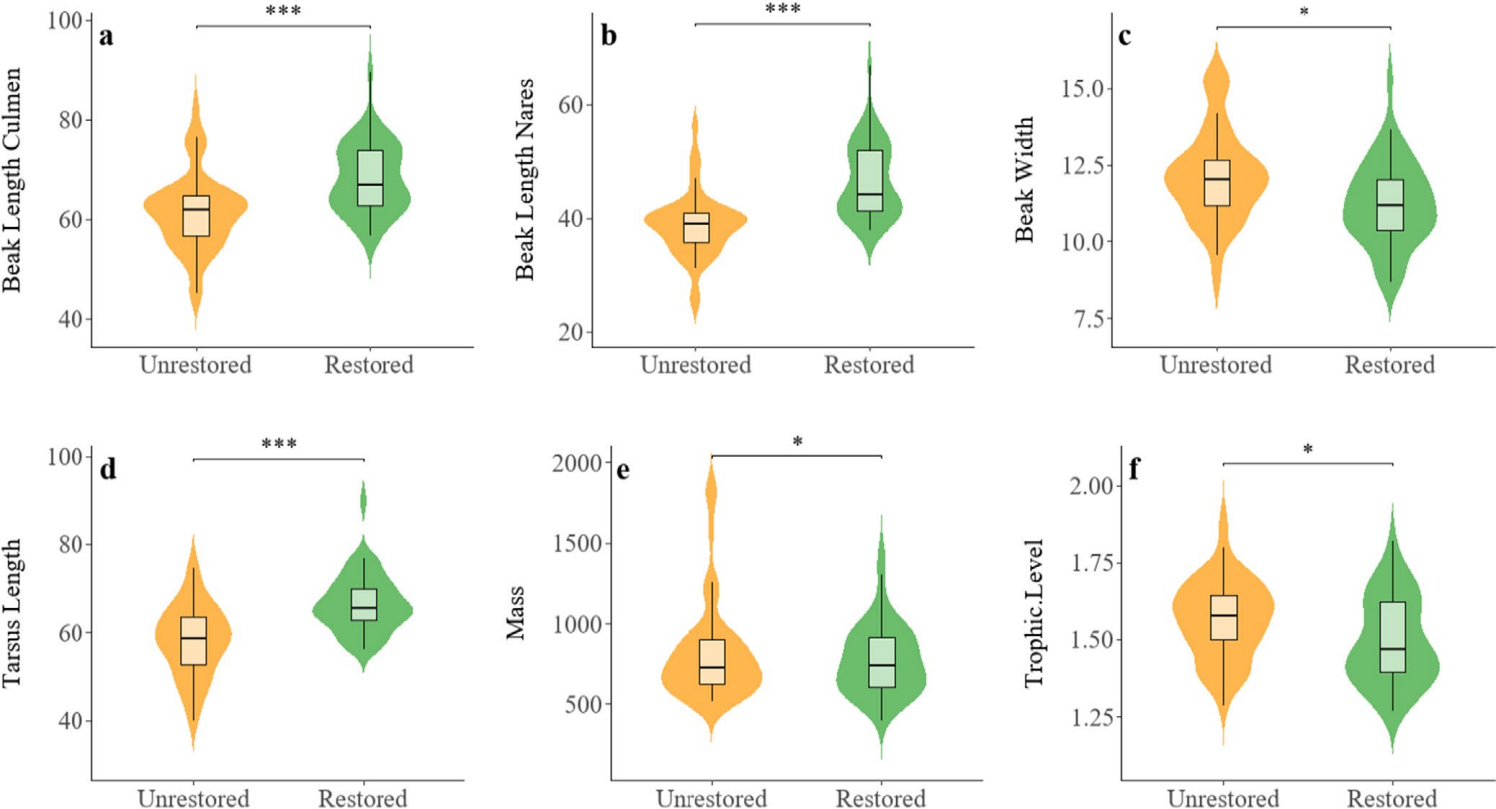
CWM showing only the cases where the LMM model indicates significant differences between wetlands, based on the weighted average (mean ± SE) between wetland types. Statistically significant values are represented by an asterisk sign: ***p < 0.001, *p < 0.05.

Eleven functional feature categories with CWM correlations > 0.6 with the PCoA axis (Table S5) were identified, including 10 morphological features and one ecological feature. LMM fitting of these 11 functional features showed that six main functional features drove the differences in wintering waterbirds between the two wetland types. They were Beak Length Culmen (ΔAIC = 14.2, *χ* ^2^ = 16.2, *p* < 0.001), Beak Length Nares (ΔAIC = 13.8, *χ* ^2^ = 17.5, *p* < 0.001), Beak Width (ΔAIC = 28.2, *χ* ^2^ = 30.2, *p* < 0.05), Tarsus Length (ΔAIC = 6.14, *χ* ^2^ = 8.1, *p* < 0.01), Mass (ΔAIC = 14.8, *χ* ^2^ = 14.5, *p* < 0.05) and Trophic Level (ΔAIC = 13.3, *χ* ^2^ = 13.5, *p* < 0.05) (Figure 3 and Table S6).

### 3.2 Patterns of Functional Beta Diversity

**FIGURE 4:**
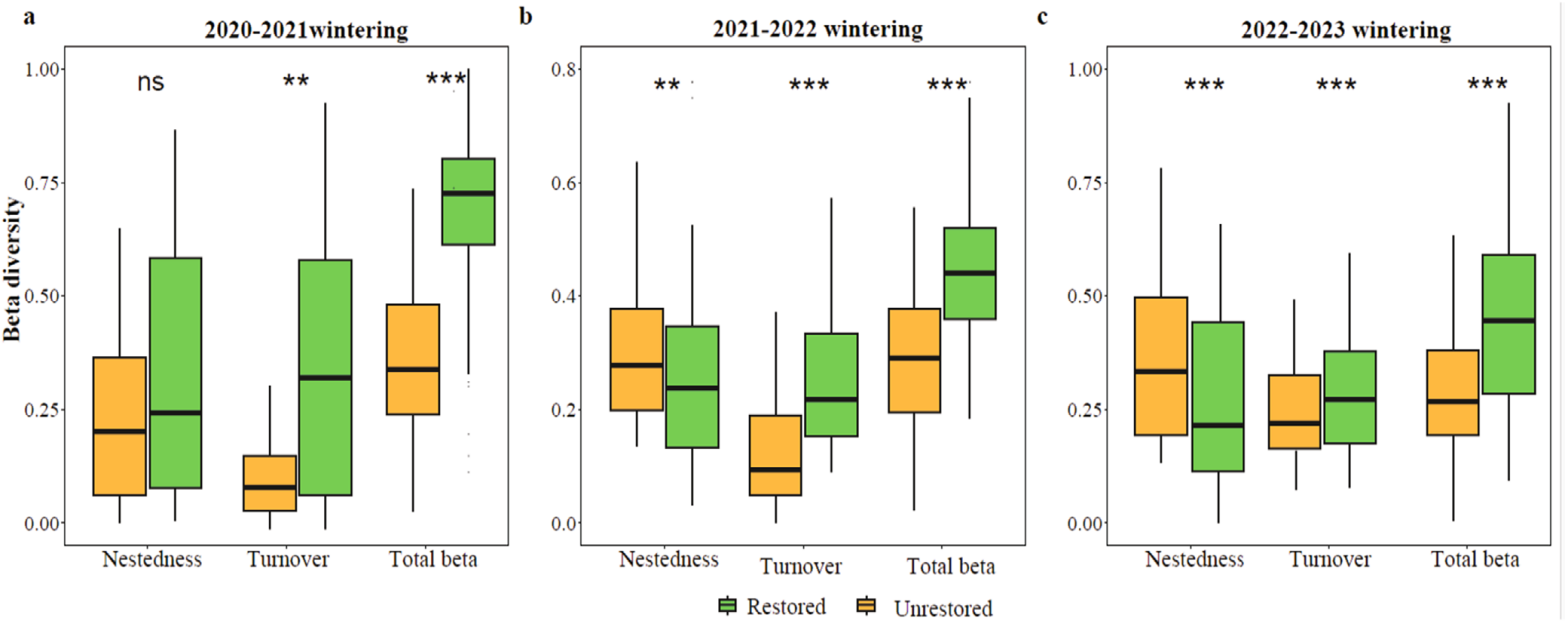
Contribution of each wetland type to regional beta diversity (as well as regional turnover and nestedness) in the gate‐controlled floodplain lake, ***p < 0.001, **p < 0.01, ns = non‐significant (*p* ≥ 0.05).

The contributions of wintering waterbirds to regional functional beta diversity and its components in the two wetland types differed significantly across different wintering years (Figure 4). As the restoration process advanced, restored wetlands exhibited higher functional turnover (*p* < 0.001) and lower functional nestedness (*p* < 0.001) compared to unrestored wetlands.

### 3.3 Factors Affecting Regional Functional Differences

**FIGURE 5:**
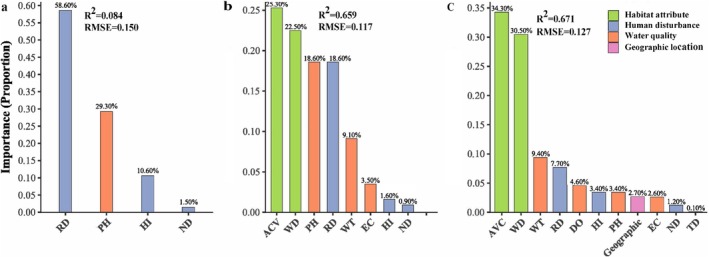
GDM results showing that environmental variables had significant explanatory power for the functional β diversity of wintering waterbirds (regional metacommunity) and its components, (a) turnover, (b) total β diversity, (c) nestedness.

GDM showed that environmental variables had significant explanatory power for the functional β diversity of wintering waterbirds and its components, with a nonlinear threshold effect. Specifically, the explanatory power of 63.4% (*R* ^2^ = 0.659, RMSE = 0.117) for total β diversity was dominated by habitat attributes—ACV (< 12% and > 60%) and water depth (WD = 50 cm as the threshold), which together explained 87.9% of the variance, followed by water quality (pH = 7.43–8.91) and anthropogenic disturbance (RD = 235 m as the threshold) both explained 18.6% (Figures 5 and Figure S2).

For nestedness components, the model achieved 61.1% explanatory power (*R* ^2^ = 0.671, RMSE = 0.127), primarily driven by habitat attributes (ACV + WD = 64.8%). High vegetation cover (ACV > 60%) and deep water conditions (WD > 220 cm) significantly exacerbated nestedness, contributing over 70% to this pattern (Figures 5, S3). In contrast, the turnover component showed lower explanatory power (8.7%; *R* ^2^ = 0.084, RMSE = 0.15), yet maintained strong influences from anthropogenic disturbance (RD) and water quality (pH), which collectively explained 87.9% of the variance through nonlinear responses. Notably, turnover rates increased in near‐disturbed areas (RD < 235 m) and decreased beyond an alkaline threshold (pH > 8.91) (Figures 5, S4).

Geographic distance exhibited minimal influence, accounting for only 2.7% of the nestedness variance. This finding was further supported by Mantel's test, which revealed no significant spatial correlation between β‐diversity of wintering waterbird communities and geographic distance (Mantel's *r* = −0.097, *p* = 0.9), suggesting that environmental filtering rather than spatial processes primarily structures the functional β‐diversity patterns in this system.

## 4 Discussion

The investigation of functional α‐ and β‐diversity components provides critical insights into restoration outcomes at local scales and the processes influencing restoration efficiency at regional levels (Coccia et al. 2021). Supporting our first hypothesis, we found that habitat type, rather than interannual seasonal variations, serves as the primary driver of functional α‐diversity differentiation at the regional scale (Figure 2). Restored wetlands demonstrated significant enhancements across multiple functional α‐diversity indices, including FRic, FDis, and FDiv. This pattern aligns closely with the “habitat complexity‐functional diversity” framework proposed by Mouillot et al. (2013). The strong positive correlation between FRic and taxonomic richness suggests that increased habitat complexity simultaneously enhances biodiversity across both taxonomic and functional dimensions (Derhé et al. 2018). Elevated FDis values indicate intensified niche differentiation within communities, where trait divergence likely reduces interspecific competition through optimized resource‐use efficiency, corroborating Villeger et al (2008)'s assertion that functional dispersion reflects resource partitioning efficiency. Furthermore, higher FDiv reflects enhanced ecological niche complementarity, facilitating the coexistence of species with complementary functional traits and contributing to the ecosystem's multifunctional stability.

Functional trait analysis further revealed the ecological superiority of restored wetlands. The divergence patterns in six pivotal functional traits—beak morphology, tarsus length, body mass, and trophic level (Figure 3)—substantiate morpho‐functional coevolution theory (Hughes et al. 2022). Variations in tridimensional beak parameters suggest that restored wetlands establish diversified feeding niches: elongated narrow beaks facilitate benthic filter‐feeding strategies, while short broad beaks optimize surface dabbling behaviors. Concurrently, differentiation in body mass and trophic levels reveals a continuum of energy allocation strategies, ranging from high‐metabolic carnivorous species to low‐energy herbivorous avifauna. This morpho‐ecological decoupling phenomenon potentially enhances community functional redundancy under environmental perturbations through compensatory mechanisms (Rosenfeld 2002). Notably, the limited temporal effects of wintering years on functional diversity likely stem from high‐precision water regulation regimes in the study area, which stabilize habitat conditions across interannual cycles.

Beta diversity provides critical insights into the mechanisms driving biodiversity change and its effect on multiple ecosystem functions, with turnover and nestedness processes representing distinct and often opposing ecological mechanisms. Distinguishing which phenomenon drives β‐diversity patterns is essential for understanding the underlying ecological processes (Baselga 2010; Bevilacqua et al. 2020). Supporting our second hypothesis, we found significant differences in the contributions of wetland types to regional functional β‐diversity and its components. As restoration progressed, restored wetlands exhibited a coupling of heightened functional turnover and reduced functional nestedness, signaling a shift in community assembly mechanisms from “environmental filtering” to “niche complementarity” (Kraft et al. 2015). The sustained intensification of functional turnover suggests that restored wetlands establish multidimensional niche gradients through microtopographic remodeling, facilitating seasonal succession of waterbird functional guilds. The markedly diminished functional nestedness indicates that restored wetlands develop unique species assemblages rather than serving as mere subsets of unrestored wetland communities. This “denesting” phenomenon may stem from anthropogenically regulated habitat patchiness, which attenuates competitive exclusion by dominant species (Tilman 1994). Additionally, this pattern may be linked to the abundance and diversity of available food resources across distinct wetland habitats. Consistent with this, turnover dominated the functional diversity of waterbirds throughout the wintering years, reflecting the unique migratory characteristics of the waterbird life cycle and the fundamental task of maximizing energy acquisition at wintering sites (Almeida et al. 2019).

Environmental filtering and dispersal limitations are widely recognized as the primary mechanisms controlling species community assembly (D'Amen et al. 2018; Jia et al. 2020). Consistent with expectations, our findings demonstrate that functional diversity differences among wetlands are driven by environmental filtering. Key habitat attributes—WD and ACV—emerged as dominant predictors of total β‐diversity and nestedness variation, exhibiting a bimodal threshold effect: the 50 cm WD threshold becomes a critical tipping point for community reorganization when ACV falls below 12% or exceeds 60%. This “window effect” aligns with Olden and Poff's (2004) bistable environmental filtering theory, where sparse vegetation zones select stress‐tolerant species through exposure risks, while densely vegetated areas filter specialists via light competition. Previous studies have noted that increased WD reduces functional group diversity (Tavares et al. 2015). In contrast, although the explanatory power of turnover components was relatively low, 87.9% of its variance stemmed from antagonistic interactions between anthropogenic disturbance (RD < 235 m) and alkaline thresholds (pH > 8.91). This likely reflects waterbird communities' responses to heterogeneous environments and the indirect effects of alkaline conditions on waterbird abundance and density by suppressing benthic invertebrate biomass (Mishra et al. 2023). Additionally, the negligible influence of geographic distance suggests that community assembly in this watershed follows an environment‐dominated species sorting paradigm rather than dispersal limitation mechanisms. This supports the metacommunity theory prediction that “environmental filtering > dispersal” (Baselga 2010; Henry and Cumming 2016). While the results underscore the importance of environmental filtering in shaping the functional diversity of wintering waterbird communities, its explanatory power (~60%) contrasts with taxonomic diversity patterns and may reflect limitations in measured variables. Future studies should further explore these dynamics, which will guide the direction of our future studies.

## 5 Conclusions

Functional diversity metrics provide critical mechanistic insights into the relationships between biodiversity and ecological functions in restored habitats. This study conducted a systematic assessment of the enhanced effects of the lakeside of Chaohu Lake restoration on wintering waterbird functional diversity through an integrated framework combining functional traits and diversity analysis. Three hypotheses were validated: conclusively showing that wetland restoration promoted functional diversity (across both α‐ and β‐diversity dimensions) among wintering waterbirds in the lakeside of Chaohu Lake.

At regional scale, functional diversity components were primarily driven by environmental filtering through trait‐based selection mechanisms. Restoration initiatives effectively mitigated the strong environmental filtering effects characteristic of unrestored wetlands on regional community assembly, underscoring the tight coupling between functional traits and environmental factors. However, it is crucial to note that these conclusions are particularly relevant to restoration scenarios in long‐term anthropogenically homogenized habitats. In naturally complex wetland systems, such as unregulated natural floodplains, the potential for functional diversity enhancement through restoration measures may be more limited. This limitation highlights the urgent need for cross‐ecosystem comparative studies to validate and generalize these findings across different wetland types and restoration contexts.

## Author Contributions

**Shanshan Xia:** conceptualization (equal), data curation (equal), formal analysis (equal), investigation (equal), methodology (equal), visualization (equal), writing – original draft (equal). **Xianglin Ji:** data curation (equal), formal analysis (equal), investigation (equal), methodology (equal), visualization (equal). **Lei Meng:** investigation (equal), methodology (equal), visualization (equal). **Lizhi Zhou:** conceptualization (equal), funding acquisition (equal), project administration (equal), supervision (equal), writing – review and editing (equal).

## Conflicts of Interest

The authors declare no conflicts of interest.

## Supporting information


Appendix S1.

Data S1.

## Data Availability

The data supporting the findings of this study are openly available in the GitHub repository https://github.com/Xianglin‐Ji/Code‐and‐Data/issues/2#issue‐2907442415.
