# Supplementary material for: Functional Diversity of Wintering Waterbird Enhanced by Restored Wetland in the Lakeshore of Chaohu Lake
Source: Ecol Evol. 2025 Jul 7;15(7):e71751. doi: 10.1002/ece3.71751 (PMC12236077; doi:10.1002/ece3.71751)
Supplement: Supplementary file 1 — Appendix S1. [file ECE3-15-e71751-s002.docx]

**SUPPORTING INFORMATION**

**Functional diversity of wintering waterbird enhanced by restored wetland in the lakeshore of Chaohu Lake**

Shanshan Xia, Xianglin Ji, Lei Meng, Lizhi Zhou^⁎^

⁎ Corresponding author: Lizhi Zhou ([zhoulz@ahu.edu.cn](mailto:zhoulz@ahu.edu.cn))


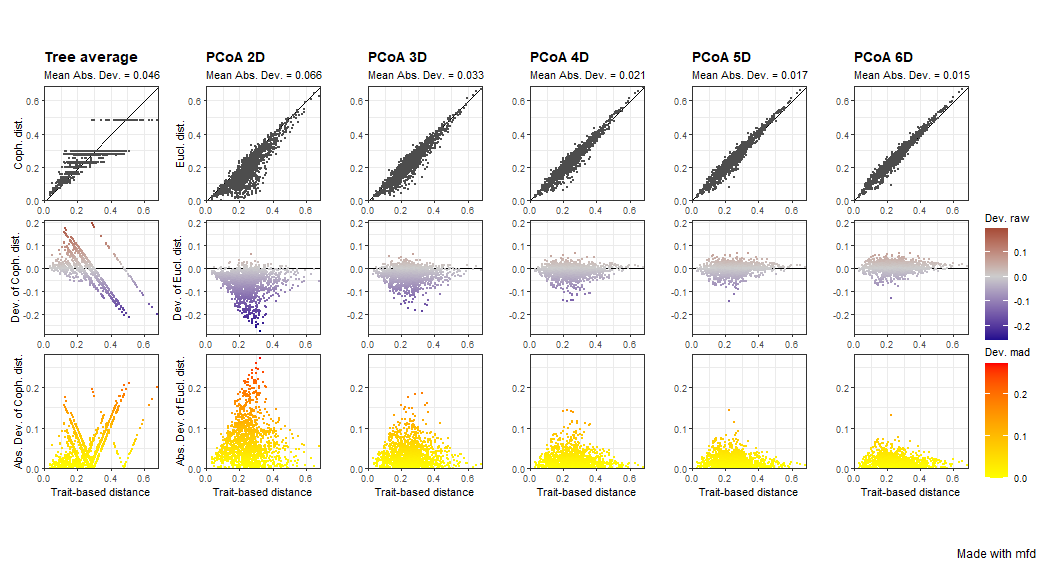


**Fig. S1 functional space quality map**

# Table S1. List of taxa collected in the study sites and taxonomic level used for trait attribution

| **Order** | **Family** | **Species** |
| --- | --- | --- |
| Anseriformes | Anatidae | *Cygnus columbianus* |
|  |  | *Anser anser* |
|  |  | *Anser cygnoid* |
|  |  | *Anser fabalis* |
|  |  | *Anser albifrons* |
|  |  | *Mergellus albellus* |
|  |  | *Tadorna ferruginea* |
|  |  | *Aythya ferina* |
|  |  | *Aythya baeri* |
|  |  | *Aythya nyroca* |
|  |  | *Aythya fuligula* |
|  |  | *Spatula querquedula* |
|  |  | *Spatula clypeata* |
|  |  | *Mareca falcata* |
|  |  | *Mareca strepera* |
|  |  | *Mareca penelope* |
|  |  | *Anas poecilorhyncha* |
|  |  | *Anas platyrhynchos* |
|  |  | *Anas acuta* |
|  |  | *Anas crecca* |
| Podicipediformes | Podicipedidae | *Tachybaptus ruficollis* |
|  |  | *Podiceps cristatus* |
|  |  | *Podiceps nigricollis* |
| Gruiformes | Rallidae | *Rallus indicus* |
|  |  | *Zapornia akool* |

**Appendix Table S1. List of taxa collected in the study sites and taxonomic level used for trait attribution**

| **Order** | **Family** | **Species** |
| --- | --- | --- |
|  |  | *Gallinula chloropus* |
|  |  | *Fulica atra* |
| Ciconiiformes | Ciconiidae | *Ciconia boyciana* |
| Pelecaniformes | Threskiornithidae | *Platalea leucorodia* |
|  | Ardeidae | *Botaurus stellaris* |
|  |  | *Nycticorax nycticorax* |
|  |  | *Ardeola bacchus* |
|  |  | *Bubulcus ibis* |
|  |  | *Ardea cinerea* |
|  |  | *Ardea purpurea* |
|  |  | *Ardea alba* |
|  |  | *Ardea intermedia* |
|  |  | *Egretta garzetta* |
| Suliformes | Phalacrocoracidae | *Phalacrocorax carbo* |
| Charadriiformes | Recurvirostridae | *Recurvirostra avosetta* |
|  |  | *Himantopus himantopus* |
|  | Charadriidae | *Charadrius placidus* |
|  |  | *Charadrius dubius* |
|  |  | *Charadrius alexandrinus* |
|  |  | *Vanellus vanellus* |
|  |  | *Vanellus cinereus* |
|  | Scolopacidae | *Limosa limosa* |
|  |  | *Calidris alpina* |
|  |  | *Gallinago gallinago* |
|  |  | *Actitis hypoleucos* |
|  |  | *Tringa ochropus* |
|  |  | *Tringa erythropus* |

# Table S2. Environmental variables used for analyses, and their definitions

| **Variables** | **abbreviation** | **Definition** |
| --- | --- | --- |
| Potential for hydrogen | pH | measured with a portable water quality analyser (Hach HQ40d, USA) in the field |
| Dissolved oxygen | DO | measured with a portable water quality analyser (Hach HQ40d, USA) in the field |
| Transparency | TD | the depth at which the white disk (Secchi disk) disappeared from the surface observer’s view |
| Water temperature | WT | measured with a portable water quality analyser (Hach HQ40d, USA) in the field |
| Electrical conductivity, | EC | measured with a portable water quality analyser (Hach HQ40d, USA) in the field |
| Water depth | WD | The actual measurement of the graduated cord |
| Aquatic vegetation cover | AVC | Visual interpretation of vegetation cover within the sampling area |
| Distance from roads | RD | Geographic distance to the nearest road measured from the center of the sampling point |
| Distance from villages | VD | Geographic distance to the nearest village measured from the center of the sampling point |
| Human interference | HI | Reflects the intensity of anthropogenic activity within the range of the sampling point and is categorized into 5 categories, 1=no disturbance, 2=low disturbance, 3=moderate disturbance, 4=more severe disturbance, and 5=strong disturbance. |

# Table S3. Functional traits

|  | **Variable** | **Description** |
| --- | --- | --- |
| **1** | Beak.Length_Culmen | Length from the tip of the beak to the base of the skull |
| **2** | Beak.Length_Nares | Length from the anterior edge of the nostrils to the tip of the beak |
| **3** | Beak.Depth | Depth of the beak at the anterior edge of the nostrils |
| **4** | Beak.Width | Width of the beak at the anterior edge of the nostrils |
| **5** | Tarsus.Length | Length of the tarsus from the posterior notch between tibia and tarsus, to the end of the last scale of acrotarsium (at the bend of the foot) |
| **6** | Wing.Length | Length from the carpal joint (bend of the wing) to the tip of the longest primary on the unflattened wing |
| **7** | Kipps.Distance | Length from the tip of the first secondary feather to the tip of the longest primary |
| **8** | Secondary | Length from the carpal joint (bend of the wing) to the tip of the first secondary, i.e. the outermost secondary adjacent to the innermost primary feather. Secondary1 is roughly equivalent to Wing length minus Kipp’s distance (measured in a fully folded and flat wing) |
| **9** | Tail.Length | Distance between the tip of the longest rectrix and the point at which the two central rectrices protrude from the skin, typically measured using a ruler inserted between the two central rectrices |
| **10** | Hand-Wing.Index | where DK is Kipp’s distance and Lw is wing length (i.e., Kipp’s distance corrected for wing size). |
| **11** | Mass | Body mass given as species average (incorporating both male and female body mass) |
| **12** | Habitat | Desert (= drylands and other open arid habitats, often sandy with very sparse vegetation);  Rock (= rocky substrate typically with no or very little vegetation, including rocky outcrops, rocky coastlines, arid stony steppes, rocky mountaintops and mountain slopes);  Grassland (= open dry to moist grass-dominated landscapes, at all elevations);  Shrubland (= low stature bushy habitats, included thornscrub, thorny or arid savanna, caatinga, xerophytic shrubland and coastal scrub);  Woodland (= medium stature tree-dominated habitats, including Acacia woodland, riparian woodlands, mangrove forests, forest edges, also more open parkland with scattered taller trees); Forest (= tall tree-dominated vegetation with more or less closed canopy, including palm forest);  Human modified (urban landscapes, intensive agriculture, gardens);  Wetland (= wide range of freshwater aquatic habitats including lakes, marshes, swamps and reedbeds);  Riverine (= associated with rivers and streams at all elevations);  Coastal (= intertidal zones within immediate vicinity of beaches, estuaries, brackish to salty marshes, including mudflats, lagoons, alkaline wetlands, coastal dunes and harbours); Marine (= pelagic, on sea near coasts, including species in the intertidal zone on beaches, and those pelagic species nesting near the sea on cliffs, islets and islands). |
| **13** | Habitat.Density | 1 = Dense habitats. Species primarily lives in the lower or middle storey of forest, or in dense thickets, dense shrubland etc. 2 = Semi-open habitats. Species primarily lives in open shrubland, scattered bushes, parkland, low dry or deciduous forest, thorn forest.  3 = Open habitats. Species primarily lives in desert, grassland, open water, low shrubs, rocky habitats, seashores, cities. Also applies to species living mainly on top of forest canopy (i.e. mostly in the open) |
| **14** | Migration | 1 = Sedentary.  2 = Partially migratory, i.e. minority of population migrates long distances, or most of population undergoes short-distance migration, nomadic movements, distinct altitudinal migration, etc. 3 = Migratory, i.e. majority of population undertakes long-distance migration |
| **15** | Trophic.Level | Herbivore = species obtaining at least 70% of food resources from plants; Carnivore = species obtaining at least 70% of food resources by consuming live invertebrate or vertebrate animals; Scavenger = species obtaining at least 70% of food resources from carrion or refuse; Omnivore = species obtaining resources from multiple trophic level in roughly equal proportion |
| **16** | Trophic.Niche | Frugivore = species obtaining at least 60% of food resources from fruit;  Granivore = species obtaining at least 60% of food resources from seeds or nuts;  Nectarivore = species obtaining at least 60% of food resources from nectar;  Herbivore = species obtaining at least 60% of food resources from other plant materials in non-aquatic systems, including leaves, buds, whole flowers etc.;  Herbivore aquatic = species obtaining at least 60% of food resources from plant materials in aquatic systems, including algae and aquatic plant leaves;  Invertivore = species obtaining at least 60% of food resources from invertebrates in terrestrial systems, including insects, worms, arachnids, etc.;  Vertivore = species obtaining at least 60% of food resources from vertebrate animals in terrestrial systems, including mammals, birds, reptiles etc.;  Aquatic Predator = species obtaining at least 60% of food resources from vertebrate and invertebrate animals in aquatic systems, including fish, crustacea, molluscs, etc;  Scavenger = species obtaining at least 60% of food resources from carrion, offal or refuse; Omnivore = Species using multiple niches, within or across trophic levels, in relatively equal proportions |
| **17** | Min.Latitude | The minimum latitudinal extent of the species range (restricted to breeding and resident range) |
| **18** | Max.Latitude | The maximum latitudinal extent of the species range (restricted to breeding and resident range) |
| **19** | Centroid.Latitude | The geometric centre of the species range (restricted to breeding and resident range). |
| **20** | Centroid.Longitude | The geometric centre of the species range (restricted to breeding and resident range) |
| **21** | Range.Size | The total area of the mapped range of the species (not the Extent of Occurrence). |

**Table S4. Fitted linear mixed models for indices of functional alpha diversity using wetland type (restored and unrestored ) as explanatory factor and overwintering years as random factor. Intercept represents unrestored wetland. Functional space was constructed considering all wintering years and wetland types.**

|  | **predictors** | **Estimates** | ***CI*** | ***P*** | **Marginal R^2^ / Conditional R^2^** | **Cohen's d** | **χ²** | **∆AIC** |
| --- | --- | --- | --- | --- | --- | --- | --- | --- |
| FDis | Intercept | 1.08 | 1.04 – 1.13 | <0.001 | 0.173 / 0.176 | 0.91 | 17.5 | **13.8** |
|  | Restored | 0.14 | 0.07 – 0.20 | <0.001 |  |  |  |  |
| FDiv | Intercept | 0.81 | 0.80 – 0.82 | <0.001 | 0.184 / 0.196 | 0.61 | 5.6 | **15.7** |
|  | Restored | 0.02 | 0.01 – 0.03 | 0.032 |  |  |  |  |
| FRic | Intercept | 4.32 | 3.61 – 5.02 | <0.001 | 0.219 / 0.232 | 1.05 | 22.3 | **18.7** |
|  | Restored | 2.19 | 1.29 – 3.08 | <0.001 |  |  |  |  |
| FEve | Intercept | 0.72 | 0.69 – 0.74 | <0.001 | 0.071 / 0.134 | 0.55 | 4 | 4.6 |
|  | Restored | 0.03 | 0.01 – 0.06 | 0.061 |  |  |  |  |

# Table S5. Pearson correlation coefficients between trait category values and PCoA axes describing community functional space with coefficients above 0.6 shown in bold

| **Functional traits** | **Correlation coefficients with PCOA axes** |
| --- | --- |
| CWM.Beak.Length_Culmen | **0.916** |
| CWM.Beak.Length_Nares | **0.952** |
| CWM.Beak.Width | **0.903** |
| CWM.Beak.Depth | **0.941** |
| CWM.Tarsus.Length | **0.821** |
| CWM.Wing.Length | **0.925** |
| CWM.Kipps.Distance | **0.977** |
| CWM.Secondary1 | **0.827** |
| CWM.Hand.Wing.Index | 0.364 |
| CWM.Tail.Length | **0.84** |
| CWM.Mass | **0.974** |
| CWM.Habitat | 0.135 |
| CWM.Habitat.Density | 0.357 |
| CWM.Migration | 0.342 |
| CWM.Trophic.Level | **0.911** |
| CWM.Trophic.Niche | -0.157 |
| CWM.Min.Latitude | 0.4 |
| CWM.Max.Latitude | 0.362 |
| CWM.Centroid.Latitude | 0.468 |
| CWM.Centroid.Longitude | -0.568 |
| CWM.Range.Size | 0.032 |

# Table S6. Fitted linear mixed models for Community Weighted Means (CWM) of functional traits for which correlations with PCoA axes were above 0.6, using wetland type (restored or unrestored) as explanatory factor and wintering years as random factor. Intercept represents unrestored wetlands.

|  | **predictors** | **Estimates** | **CI** | ***P*** | **Marginal R2 / Conditional R2** | **Cohen's d** | **χ²** | **∆AIC** |
| --- | --- | --- | --- | --- | --- | --- | --- | --- |
| **Beak Length Culmen** | Intercept | 61.55 | 59.01 – 64.09 | <0.001 | 0.175 / 0.192 | 0.9 | 16.22 | **14.217** |
|  | Restored | 6.71 | 3.56 – 9.86 | **<0.001** |  |  |  |  |
| **Beak Length Nares** | Intercept | 38.78 | 36.54 – 41.03 | <0.001 | 0.299 / 0.322 | 1.28 | 30.21 | **28.21** |
|  | Restored | 7.92 | 5.32 – 10.53 | **<0.001** |  |  |  |  |
| **Beak Width** | Intercept | 12.14 | 11.70 – 12.58 | <0.001 | 0.163 / 0.171 | 0.63 | 8.14 | **6.14** |
|  | Restored | -0.91 | -1.53 – -0.29 | **0.004** |  |  |  |  |
| Beak.Depth | Intercept | 13.39 | 12.98 – 13.80 | <0.001 | 0.084 / 0.091 | 0.58 | 6.91 | 4.9 |
|  | Restored | -0.78 | -1.36 – -0.20 | 0.09 |  |  |  |  |
| **Tarsus Length** | Intercept | 58.11 | 55.33 – 60.88 | <0.001 | 0.249 / 0.279 | 1.13 | 24.57 | **22.57** |
|  | Restored | 8.42 | 5.29 – 11.55 | **<0.001** |  |  |  |  |

**Appendix Table S6. Fitted linear mixed models for Community Weighted Means (CWM) of functional traits for which correlations with PCoA axes were above 0.6, using wetland type (restored or unrestored) as explanatory factor and wintering years as random factor. Intercept represents unrestored wetlands.**

|  | **predictors** | **Estimates** | **CI** | ***P*** | **Marginal R2 / Conditional R2** | **Cohen's d** | **χ²** | **∆AIC** |
| --- | --- | --- | --- | --- | --- | --- | --- | --- |
| Wing.Length | Intercept | 258.48 | 251.19 – 265.76 | <0.001 | 0.017 / 0.019 | 0.26 | 1.42 | 5.7 |
|  | Restored | -6.21 | -16.52 – 4.10 | 0.234 |  |  |  |  |
| Tail.Length | Intercept | 89.84 | 86.95 – 92.73 | <0.001 | 0.022 / 0.026 | 0.08 | 0.12 | -1.8 |
|  | Restored | 0.74 | -3.35 – 4.82 | 0.72 |  |  |  |  |
| **Mass** | Intercept | 823.02 | 740.47 – 905.57 | <0.001 | 0.21 / 0.24 | 1.9 | 7.5 | **12.4** |
|  | Restored | -51.08 | -167.83 – 65.67 | **0.038** |  |  |  |  |
| Kipps.Distance | Intercept | 120.44 | 116.15－124.73 | <0.001 | 0.017 / 0.021 | 8.17 | 1.32 | 6.8 |
|  | Restored | -6.21 | -16.52 – 4.10 | 0.234 |  |  |  |  |
| Secondary1 | Intercept | 140.12 | 135.83 – 144.41 | <0.001 | 0.021 / 0.032 | 0.29 | 1.77 | 0.22 |
|  | Restored | 4.09 | -1.98 – 10.15 | 0.184 |  |  |  |  |
| **Trophic Level** | Intercept | 1.56 | 1.52 – 1.61 | <0.001 | 0.26 / 0.31 | 0.53 | 5.93 | **5.93** |
|  | Restored | -0.07 | -0.13 – -0.01 | **0.015** |  |  |  |  |


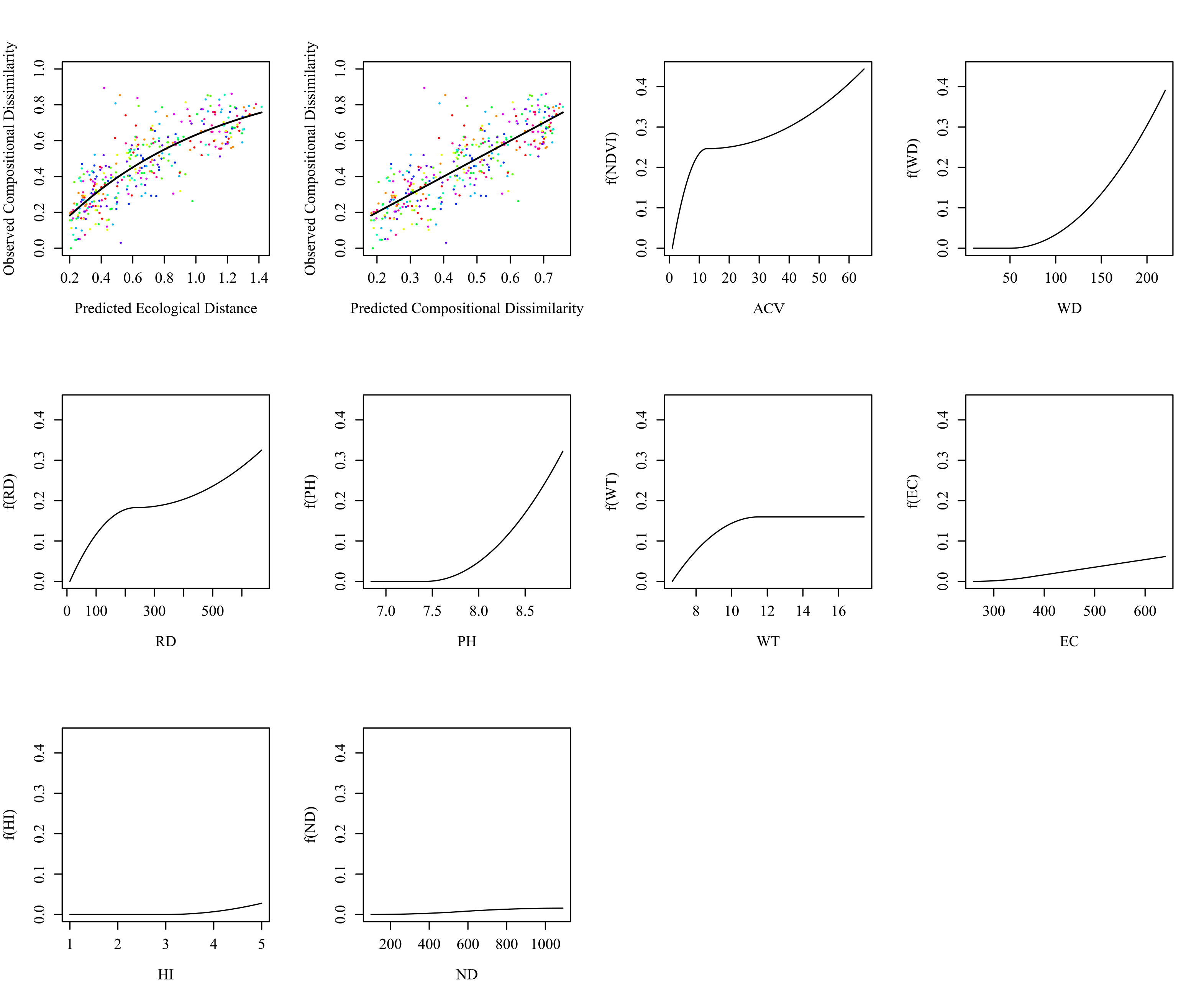


**Fig. S2. Effects of environmental and spatial variables on total β diversity in waterbird communities based on GDM results. y axis indicates the effect of each explanatory variable on β diversity. The shape of the curve depicts the effect of each explanatory variable on functional β diversity along its gradient (x axis).**


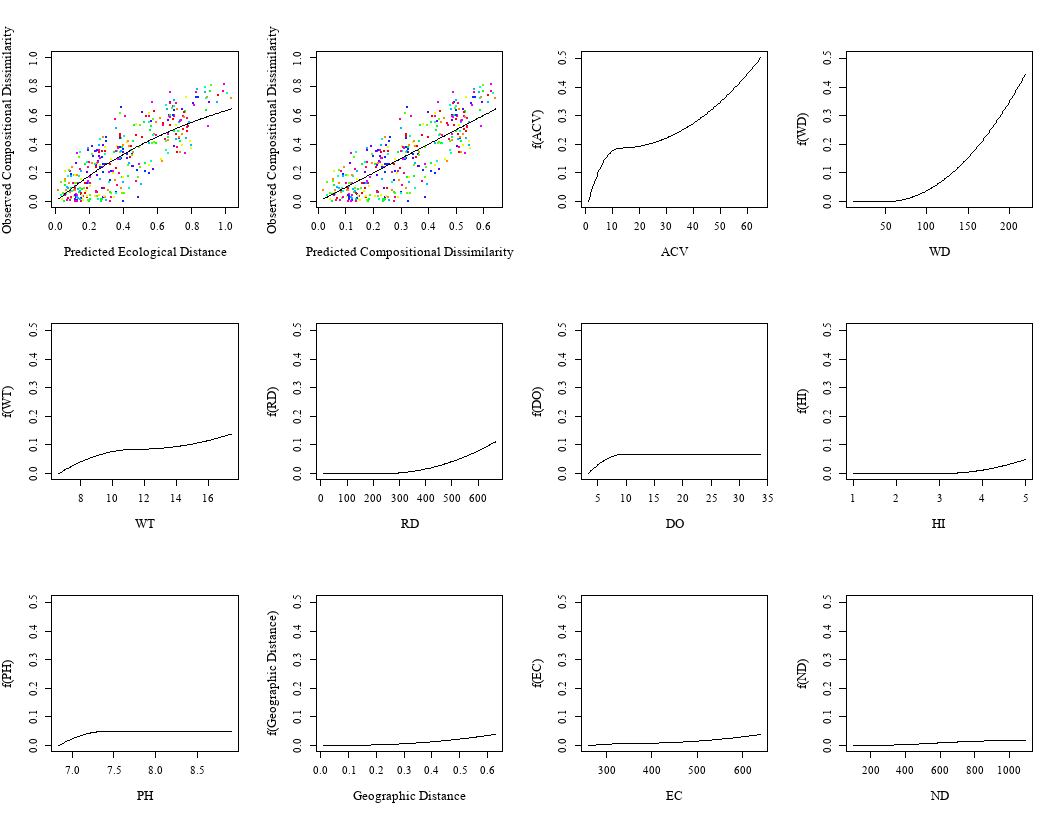


**Fig. S3. Effects of environmental and spatial variables on nestedness in waterbird communities based on GDM results. y axis indicates the effect of each explanatory variable on β diversity. The shape of the curve depicts the effect of each explanatory variable on functional β diversity along its gradient (x axis).**


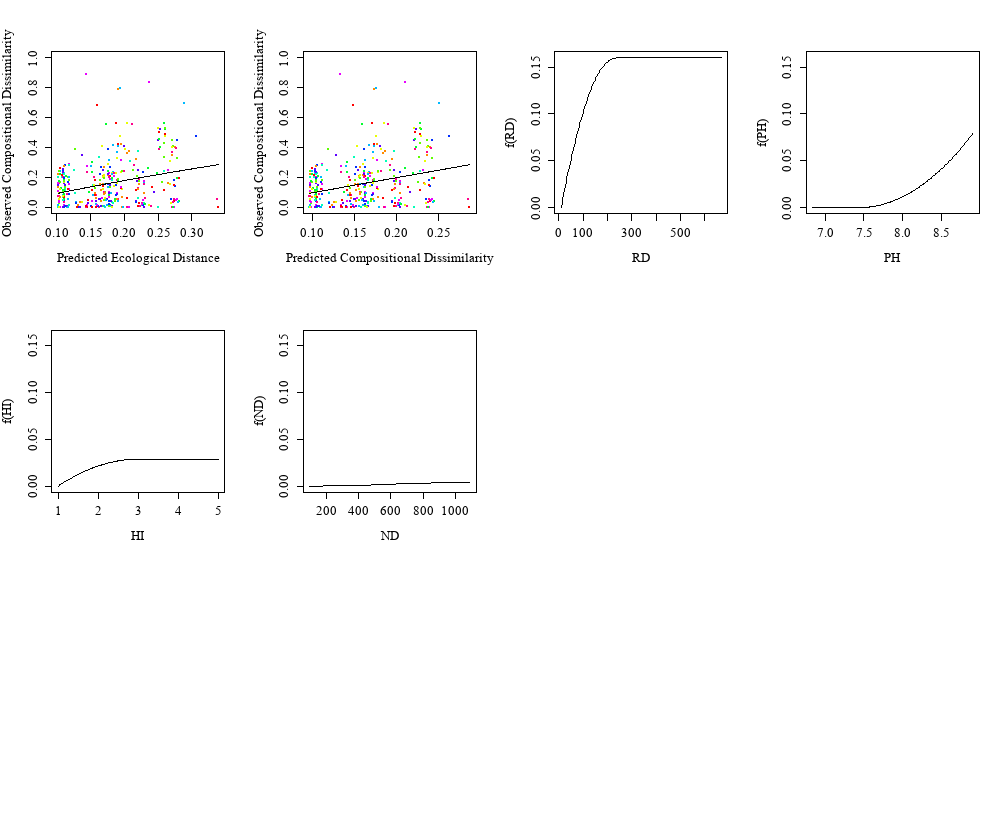


# Fig. S4. Effects of environmental and spatial variables on turnover in waterbird communities based on GDM results. y axis indicates the effect of each explanatory variable on β diversity. The shape of the curve depicts the effect of each explanatory variable on functional β diversity along its gradient (x axis).
